# Risk factor analysis and prediction model for papillary thyroid carcinoma with lymph node metastasis

**DOI:** 10.3389/fendo.2023.1287593

**Published:** 2023-10-30

**Authors:** Juerong Lu, Jintang Liao, Yunhao Chen, Jie Li, Xinyue Huang, Huajun Zhang, Bo Zhang

**Affiliations:** ^1^ Department of Ultrasonic Imaging, Xiangya Hospital, Central South University, Changsha, Hunan, China; ^2^ Department of Oncology, National Health Commission of the People's Republic of China (NHC) Key Laboratory of Cancer Proteomics, Xiangya Hospital, Central South University, Changsha, Hunan, China; ^3^ Laboratory of Structural Biology, Xiangya Hospital, Central South University, Changsha, Hunan, China; ^4^ Molecular Imaging Research Center of Central South University, Changsha, Hunan, China

**Keywords:** papillary thyroid carcinoma, lymph node metastasis, ultrasound, risk factor, nomogram

## Abstract

**Objective:**

We aimed to identify the clinical factors associated with lymph node metastasis (LNM) based on ultrasound characteristics and clinical data, and develop a nomogram for personalized clinical decision-making.

**Methods:**

A retrospective analysis was performed on 252 patients with papillary thyroid carcinoma (PTC). The patient’s information was subjected to univariate and multivariate logistic regression analyses to identify risk factors. A nomogram to predict LNM was established combining the risk factors. The performance of the nomogram was evaluated using receiver operating characteristic (ROC) curve, calibration curve, cross-validation, decision curve analysis (DCA), and clinical impact curve.

**Results:**

There are significant differences between LNM and non-LNM groups in terms of age, sex, tumor size, hypoechoic halo around the nodule, thyroid capsule invasion, lymph node microcalcification, lymph node hyperechoic area, peak intensity of contrast (PI), and area under the curve (AUC) of the time intensity curve of contrast (*P*<0.05). Age, sex, thyroid capsule invasion, lymph node microcalcification were independent predictors of LNM and were used to establish the predictive nomogram. The ROC was 0.800, with excellent discrimination and calibration. The predictive accuracy of 0.757 and the Kappa value was 0.508. The calibration curve, DCA and calibration curve demonstrated that the prediction model had excellent net benefits and clinical practicability.

**Conclusion:**

Age, sex, thyroid capsule invasion, and lymph node microcalcification were identified as significant risk factors for predicting LNM in patients with PTC. The visualized nomogram model may assist clinicians in predicting the likelihood of LNM in patients with PTC prior to surgery.

## 1 Introduction

According to the latest global cancer statistics, thyroid cancer is responsible for 586,000 cases worldwide and ranks in 9th place for incidence in 2020 with an estimated annual mortality of 44,000 (1). PTC is the most common histologic subtype of thyroid cancer and has a rapid increase in incidence globally over recent decades (2). PTC are prone to early metastasizes to lymph nodes with a reported incidence from 30 to 80% at initial diagnosis. LNM has been confirmed to be a significant risk factor affecting the survival rate of PTC patients, and is closely related to distant metastasis, recurrence, and poor prognosis (3).

Currently, surgery remains the primary treatment option for PTC, however, controversy persists regarding lymph node management (4). LNM preoperatively strongly correlates with distant metastasis, high locoregional recurrence, and enhanced death risk (5). Early implementation of appropriate surgical approaches and complete dissection of metastatic lymph nodes can reduce the probability of a second operation, and improve the prognosis and survival rate of the patient (6). Nevertheless, by reducing unnecessary prophylactic lymph node dissection, the occurrence of surgery-related complications can be minimized, such as hypoparathyroidism, recurrent laryngeal nerve damage, nerve injury to the voice, etc (7). Preoperative accurate assessment of whether there is lymph node metastasis in PTC is crucial for clinical decision-making and patient prognosis.

However, there are limited preoperative methods available for assessing LNM in PTC. US is the preferred diagnostic method for LNM with a high accuracy and sensitivity in identifying LNM (8). The additional use of contrast-enhanced ultrasound (CEUS) could provide qualitative and quantitative blood perfusion information and improve the diagnostic accuracy of thyroid nodules (9). Qualitative and quantitative Contrast-Enhanced Ultrasound analysis demonstrated a high diagnostic accuracy for the preoperative diagnosis of metastatic lymph nodes in patients with PTC. However, most sonographic features of metastatic lymph node are atypical, especially in early stages. Even under contrast-enhanced ultrasound, the early metastatic lymph nodes are not easy to detect (10). The assessment of LNM in patients with PTC before surgery presents significant difficulties and challenges for clinical radiologists.

Our study aimed to investigate risk factors associated with LNM and develop a user-friendly nomogram incorporating clinical and ultrasound features for quantitative prediction of LNM risk in PTC. The approach will aid clinicians in making informed therapeutic decisions, thereby improving patient prognosis.

## 2 Materials and methods

### 2.1 Participants

This prospective study was approved by the Ethics Committee of our Hospital (No. 202206140) and conducted in accordance with guidelines for good clinical practice. The study included 252 consecutive patients who underwent thyroidectomy and lymph node dissection for PTC at our institution from May 2019 to March 2022. Prior to surgery, all patients underwent preoperative serological examination, two-dimensional ultrasound (2D-US) and CEUS of the thyroid. The pathological results of LNM were served as the gold standard. The patients were classified into two groups based on the post-dissection pathological results of the lymph nodes. One group had lymph node metastases (LNM, n=139), while the other group did not (non-LNM, n=113).

The inclusion criteria were as follows: (a) Primary surgical resection was performed with a pathological diagnosis of PTC. (b) The patient underwent partial or total thyroidectomy and lymph node dissection as the surgical procedure. (c) Confirmation of lymph node metastasis was based on postoperative pathology; (d) Preoperative serological examination, 2D-US and CEUS of the thyroid were performed before surgery with complete clinicopathological information. (e) The date between serological examination, ultrasonography and surgery did not exceed one month.

The exclusion criteria were as follows: (a) Other types of thyroid cancer or PTC with other types of thyroid cancer was confirmed through surgical pathology. (b) History of previous neck surgery. (c) Age <18 years old.

### 2.2 Assessment of variables

The baseline clinical information of patients included age, sex (male, female). Biochemical indicators included free thyroid hormone (FT4), free triiodothyronine (FT3), hypersensitive thyroid stimulating hormone (TSH), thyroglobulin (TG), antithyroglobulin antibody (TGA), thyroid peroxidase antibody (TPOAB), parathyroid hormone (PTH), carcinoembryonic antigen (CEA). The results of FT_4_, FT_3_, TSH, TG, TGA, TPOAB, PTH, and CEA were detected within one month before surgery. According to the clinical experience, the thresholds set for FT4, FT3, TSH, TG, TGA, TPOAB, PTH, and CEA were as follows: FT4 12-22 pmol/L, FT3 2.8-7.1 pmol/L, TSH 0.27-4.2 uIU/ml, TG 3.5-77 ng/ml, TGA 0-115.00 IU/mL, TPOAB 0-34.00 IU/mL, PTH 15-65 pg/ml, CEA <5.0 ng/mL. (**Table 1** for details).

**Table 1:** Variables of patients’ clinical data in univariate analysis.

Variables		LNM	non-LNM	*P-*value	OR
Age (mean ± SD, years)		41 ± 10	46 ± 12	<0.001*	0.958
Sex (n)				0.002*	0.395
	Male	49	20		
	Female	90	93		
FT4 (n)				0.178	
	Normal	122	105		
	High	6	5		
	Low	10	2		
FT3 (n)				0.344	
	Normal	127	108		
	High	7	2		
	Low	4	2		
TSH (n)				0.689	
	Normal	119	91		
	High	15	16		
	Low	5	4		
TG (n)				0.345	
	Normal	93	79		
	High	13	5		
	Low	27	23		
TGA (n)				0.575	1.179
	Normal	100	86		
	High	37	27		
TPOAB (n)				0.834	0.938
	Normal	107	87		
	High	30	26		
PTH (n)				0.107	
	Normal	124	104		
	High	11	3		
	Low	3	6		
CEA (n)				0.295	3.254
	Normal	134	109		
	High	4	1		

*P-value < 0.05 was regarded as statistically significant.

2D-US and CEUS indicators for thyroid nodule included the following characteristics: thyroid diffuse lesions, multifocality, tumor size, location, echogenicity, shape, aspect ratio (A/T, wider and tall ratio), boundary, margin, hypoechoic halo (Substantial, not the annular vessel surrounding the nodules), posterior attenuation, calcification, blood flow, thyroid capsule invasion, enhancement level, enhancement mode, enhancement uniformity and perfusion defect (**Table 2** for details).

**Table 2:** Variables of thyroid nodule 2D-US and CEUS in univariate analysis.

Variables		LNM	non-LNM	*P-*value	OR
Thyroid diffuse lesions (n)				0.966	1.012
	Yes	36	29		
	No	103	84		
Multifocality (n)				0.454	1.223
	Multifocal	96	73		
	Unifocal	43	40		
Tumor size		12.00 ± 7.60	9.79 ± 7.85	0.031*	1.043
(mean ± SD,mm)					
Horizontal position (n)				0.976	
	Left lobe	66	55		
	Isthmus	8	6		
	Right lobe	65	52		
Vertical position (n)				0.063	
	Upper	35	26		
	Middle	46	55		
	Lower	50	26		
	Isthmus	8	6		
Echogenicity (n)				0.82	
	Hypoechoic	115	93		
	Very hypoechoic	14	8		
	Isoechoic	1	1		
	Mixed echo	2	3		
	Strong and weak echo	7	8		
Shape (n)				0.663	
	Regular	25	22		
	Slightly reguilar	35	23		
	Irregular	79	68		
A/T (n)				0.161	0.698
	≤1	86	60		
	>1	53	53		
Boundary (n)				0.536	
	Clear	51	43		
	Slightly clear	59	41		
	Fuzzy	29	29		
Margin (n)				0.381	
	Rough	76	62		
	Angulation	46	34		
	Burr	11	15		
	Differential leaf	6	2		
Hypoechoic halo				0.009*	2.364
around the nodule (n)					
	Yes	39	16		
	No	100	97		
Posterior attenuation (n)				0.858	0.931
	Yes	15	13		
	No	124	100		
Calcification (n)				0.127	
	Microcalcification	89	57		
	Coarse calcification	4	2		
	Micro-coarse calcification	13	14		
	No calcification	33	40		
Blood flow (n)				0.87	
	No blood flow	14	13		
	One-two spots of blood flow	32	30		
	Three-four spots or two strips of blood flow	41	32		
	More than two strips of	52	38		
	blood flow				
Thyroid capsule invasion (n)				<0.001*	4.04
	Yes	84	31		
	No	55	82		
Enhancement level (n)				0.062	
	Hypo-enhancement	83	78		
	Iso-enhancement	27	24		
	Hyper-enhancement	29	11		
Enhancement mode (n)				0.962	
	Diffuse enhancement	96	81		
	Centripetal enhancement	41	32		
	Centrifugal enhancement	2	0		
Enhancement uniformity (n)				0.437	0.776
	Homogeneous Enhancement	23	23		
	Heterogeneous Enhancement	116	90		
Perfusion defect (n)				0.104	1.648
	Yes	22	37		
	No	117	76		

*P-value < 0.05 was regarded as statistically significant.

2D-US indicators of lymph node included the following characteristics: lymph node morphology, microcalcification, hyperechoic change, dermatomedullary boundary and blood flow. (**Table 3** for details).

**Table 3:** Variables of lymph node 2D-US in univariate analysis.

Variables		LNM	non-LNM	*P-*value	OR
Morphology (n)				0.122	
	Regular	130	105		
	Irregular	6	5		
	Round	3	3		
Microcalcification (n)				<0.001*	10.966
	Yes	34	5		
	No	105	108		
Hyperechoic change (n)				0.031*	9.625
	Yes	7	5		
	No	132	108		
Cystic change (n)				0.286	3.319
	Yes	2	3		
	No	137	110		
Dermatomedullary boundary (n)				0.102	1.23
	Clear	133	112		
	Obscure	6	1		
Blood flow (n)				0.771	
	Normal portal blood flow	116	106		
	No blood flow	11	7		
	Rich blood flow	12	0		

*P-value < 0.05 was regarded as statistically significant.

Qualitative and quantitative Contrast-Enhanced Ultrasound analysis of thyroid nodule included Base Intensity (BI), Arrival Time (AT), Time to Peak (TTP), Peak Intensity (PI), Ascending Slope (AS), Area Under Curve (AUC). (**Table 4** for details).

### 2.3 US images acquisition and analysis

The US examination was independently performed by one of the two experienced radiologists with more than 20 years of experience in thyroid ultrasound diagnosis. All conventional US and CEUS examinations were performed by Resona R9 & 7S (Mindray Medical, Shenzhen, China) and Acuson Sequoia (Siemens, Erlangen, Germany). The ultrasound contrast agent used in thyroid contrast-enhanced ultrasound was the pure blood-pool contrast agent sulfur hexafluoride microbubble (SonoVue, Bracco, Italy).

Inclusion criteria for quantitative analysis of contrast-enhanced ultrasound of thyroid nodules: (a) CEUS recording lasted longer than one minute. (b) Thyroid nodule appeared in the observation screen completely. (c) Thyroid nodules were not significantly displaced.

Quantitative analysis of contrast-enhanced ultrasound: the region of interest (ROI) was delineated along the edge of the thyroid nodule, and the ROI was delineated no less than three times. Finally, the highest goodness of fit (GOF) was selected.

### 2.4 Statistical analysis

Statistical analysis was performed using SPSS 22 software and R software. Univariate and multivariate analyses were conducted for screening risk factors significantly associated with LNM. The statistically significant variables in the univariate analysis were subsequently included in the multivariate logistic regression analysis to construct a risk prediction model-nomogram. The discriminative power and consensus degree of our predictive model were assessed using the receiver operating characteristic (ROC) curve and the calibration curve. The training cohort (70% of patients) and the validation cohort (30% of patients) were randomly sampled for cross-validation. were used to evaluate The consistency of the results was assessed using the accuracy and Kappa values. Decision curve analysis (DCA) and clinical impact curve were applied to assess the feasibility and application value of the model in clinical practice.

## 3 Results

### 3.1 Patients’ clinical data univariate analysis

A total of 252 patients with PTC were enrolled in this study. They were divided into LNM and non-LNM groups in accordance with LNM or not. In the LNM group, there were 90 female and 49 male patients. The age of the patients ranged from 18 to 63 years, with a mean age of 41 ± 10 years. In the non-LNM group, there were 93 female and 20 male patients. The age of the patients ranged from 22 to 70 years, with a mean age of 46 ± 12 years.

At univariate analysis, age (OR=0.958, *P*<0.001), sex (OR=0.395, *P*=0.002) were found as the potential risk factors associated with LNM in PTC patients. However, the *p* values of FT4, FT3, TSH, TG, TGA, TPOAB, PTH, and CEA were all higher than 0.05, and there was no significant difference between the two groups (**Table 1**).

### 3.2 Thyroid nodule 2D-US and CEUS univariate analysis

At univariate analysis, tumor size (OR=1.043, *P*=0.031), hypoechoic halo around the nodule (OR=2.364, *P*=0.009), thyroid capsule invasion (OR=4.040, *P*<0.001) were found as the potential risk factors associated with LNM in PTC patients (**Table 2**).

### 3.3 Lymph node 2D-US univariate analysis

At univariate analysis, lymph node microcalcification (OR=10.966, *P*<0.001), lymph node hyperechoic change (OR=9.625, *P*=0.031) were found as the potential risk factors associated with LNM in PTC patients (**Table 3**).

### 3.4 Qualitative and quantitative CEUS analysis of thyroid nodule univariate analysis

Quantitative analysis results will be affected by patient’s cough, speech, swallowing or other reasons which will lead to a significant displacement of the lesion. There were some of the videos that were less than a minute tall. Therefore, a total of 151 patients with PTC were included in the final quantitative analysis, including 82 patients with LNM (54%) and 69 patients without LNM (46%).

At univariate analysis, PI (OR=0.954, *P*=0.029), AUC (OR=0.999, *P*=0.011) were found as the potential risk factors associated with LNM in PTC patients (**Table 4**).

**Table 4:** Variables of qualitative and quantitative CEUS analysis in univariate analysis.

Variables	LNM	non-LNM	*P-*value	OR
BI (mean ± SD)	5.99 ± 4.64	7.24 ± 5.99	0.154	0.956
AT (mean ± SD)	2.78 ± 3.69	2.71 ± 3.26	0.154	0.956
TTP (mean ± SD)	16.71 ± 5.82	17.05 ± 5.09	0.703	0.989
PI (mean ± SD)	27.13 ± 8.09	30.08 ± 7.93	0.029*	0.954
AS (mean ± SD)	1.28 ± 0.50	1.20 ± 0.32	0.284	1.524
AUC (mean ± SD)	1096.64 ± 466.16	1317.45 ± 540.54	0.011*	0.999

*P-value < 0.05 was regarded as statistically significant.

### 3.5 Multivariate logistic regression analyses

The nine variables selected by univariate analysis (age, sex, size, hypoechoic halo around the nodule, thyroid capsule invasion, lymph node microcalcification, lymph node hyperechoic change, PI and AUC were entered into the regression model for multivariate logistic regression analysis. The results showed that age (*P*=0.024), sex (*P*=0.021), thyroid capsule invasion (*P*<0.001), and lymph node microcalcification (*P*=0.005) were found as risk factors highly associated with lymph node metastasis in PTC (**Table 5**).

**Table 5:** Nine variables selected by univariate analysis in multivariate analysis.

Variables	B	S.E.	W	Multivariate OR (95% CI)	*P-*value
Age	-1.037	0.458	5.127	0.355 (0.145-0.870)	0.024*
Sex	-0.044	0.019	5.367	0.957 (0.922-0.993)	0.021*
Tumor size	-0.017	0.026	0.414	0.983 (0.934-1.035)	0.52
hypoechoic halo	0.673	0.509	1.748	1.960 (0.723-5.314)	0.186
thyroid capsule invasion	1.627	0.439	13.717	5.086 (2.151-12.030)	<0.001*
LN microcalcification	2.009	0.72	7.786	7.456 (1.818-30.576)	0.005*
Hyperechoic change	1.949	1.166	2.796	7.022 (0.715-68.972)	0.095
PI	0.019	0.038	0.241	1.019 (0.946-1.097)	0.623
AUC	-0.001	0.001	2.274	0.999 (0.998-1.000)	0.132

*P-value < 0.05 was regarded as statistically significant.

### 3.6 Development of prediction model and nomogram for predicting LNM in PTC patients

Based on the results of the multivariate logistic regression analysis, the logistic prediction model was developed as follows: logit (*P*) = 2.380-1.037* sex-0.044 * age +1.627* thyroid capsule invasion +2.009* lymph node microcalcification (0 for male, 1 for female; 0 for no thyroid capsule invasion, 1 for thyroid capsule invasion; 0 for no lymph node microcalcification, 1 for lymph node microcalcification).

**Figure 1:**
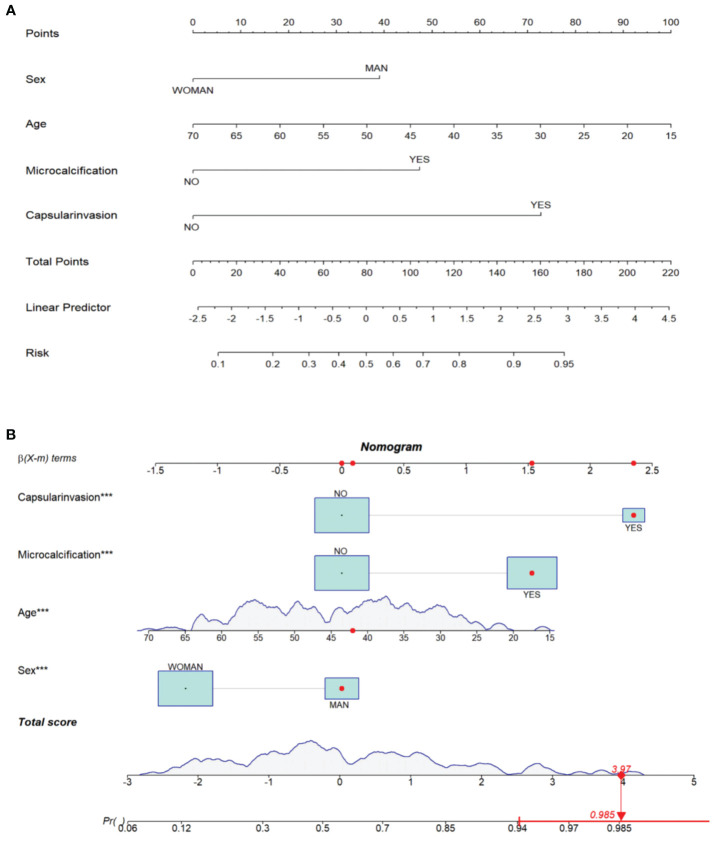
**(A)** Concise nomogram for predicting LNM in PTC patients. **(B)** Dynamic nomogram for predicting LNM in PTC patients.

**Figure 2:**
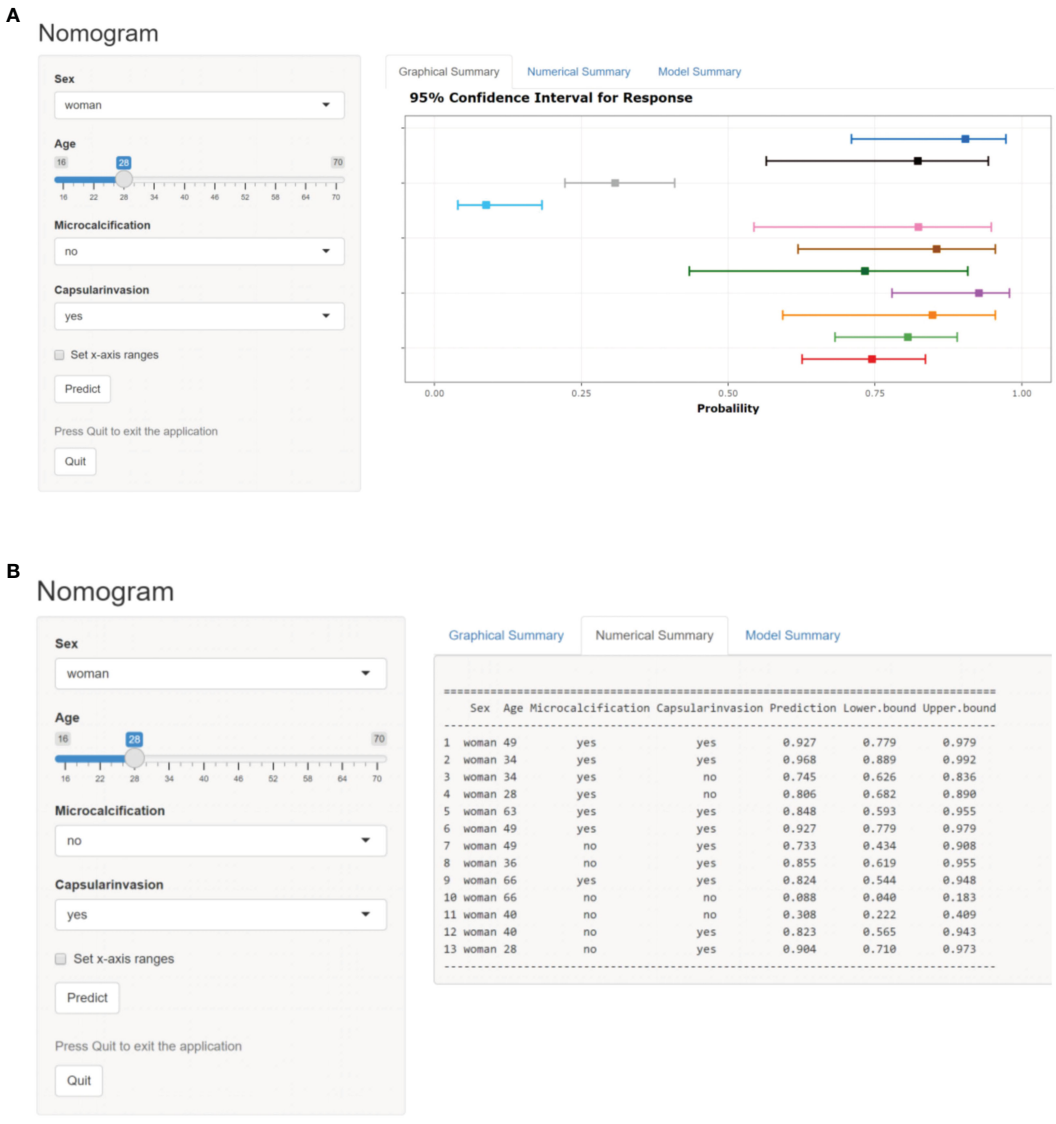
**(A)** Dynamic web-based nomogram for predicting LNM in PTC patients (screen shot 1). **(B)** Dynamic web-based nomogram for predicting LNM in PTC patients (screen shot 2).

In order to facilitate the clinical application and make the model become convenient and visual, we use R software and “rms” software package to construct the nomogram and make various forms of nomogram. Finally, we apply and register the web nomogram: https://juenomogram.shinyapps.io/DynNomapp/ (**Figures 1**, **2**).

### 3.7 Evaluation of clinical prediction model

**Figure 3:**
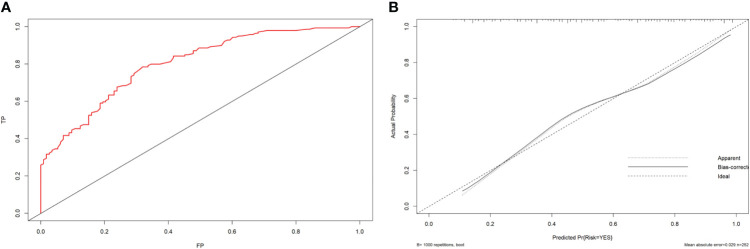
**(A)** ROC curve analysis to predict LNM in PTC patients. **(B)** Calibration curve of the nomogram. The calibration curve illustrates the calibration of the nomogram in terms of the agreement between the predicted risk of LNM and the observed outcomes of LNM. The 45° dotted gray line represents a perfect prediction, and the solid black line represents the predictive performance of the nomogram. The solid line has a closer fit to the dotted line, which indicates better predictive accuracy of the nomogram.

The ROC curve was drawn by R language. The nomogram yielded the AUC (0.800; 95% CI: 0.747-0.853, *p* < 0.001) which indicated that the prediction model was good and had good clinical predictive ability.The calibration curve was drawn by bootstrap 1000 resampling method. The calibration curve showed that a mostly agreement between the predicted and actual results of the nomogram model, indicating that the calibration degree of the model was good (**Figure 3**).

### 3.8 Validation of clinical prediction model

The R language was used for operation. 176 cases (70%) were selected as the training set, 76 cases (30%) were selected as the validation set. The number of iterations was 1. The results were: accuracy =0.757, Kappa=0.508.

The accuracy is greater than 0.7, indicating that the prediction result is good. Kappa is used to measure the stability of the model. The higher the Kappa value, the better the consistency. When the Kappa value is between 0.4 and 0.6, the consistency is acceptable.

### 3.9 Evaluation of clinical decision-making ability and practical application

**Figure 4:**
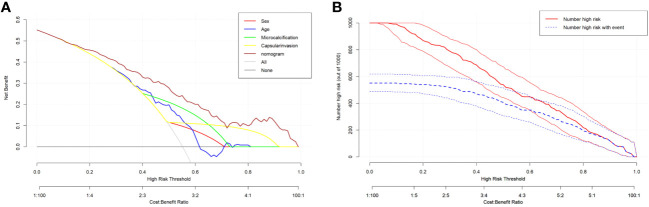
**(A)** Decision curve analysis for the nomogram. The y-axis measures the net benefit. The x-axis measures the threshold probability. **(B)** Clinical impact curve of prediction model.

**Figure 5:**
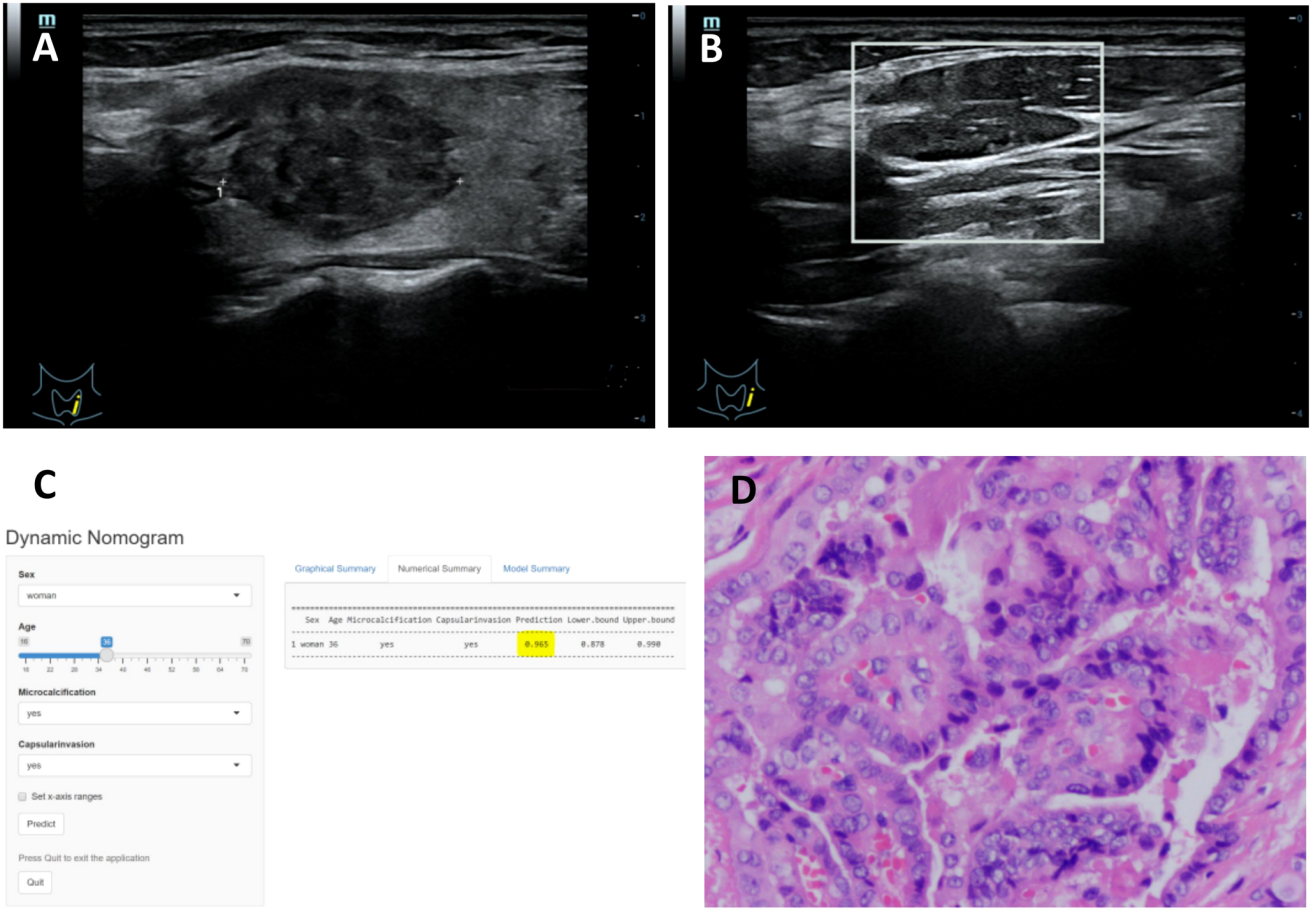
**(A–D)** Title: Representative example of dynamic web-based nomogram 520 prediction for a 36-year-old female PTC patient with LNM. **(A)** US images showed the maximum diameter of hypoechoic thyroid nodule was 23.8mm in left lobe with thyroid capsule invasion. **(B)** US images showed there was microcalcification in the left lymph node. **(C)** Dynamic web-based nomogram predicted that the risk of LNM in this patient was 0.965. **(D)** Pathology showed PTC in left lobe with thyroid capsule invasion and LNM.

**Figure 6:**
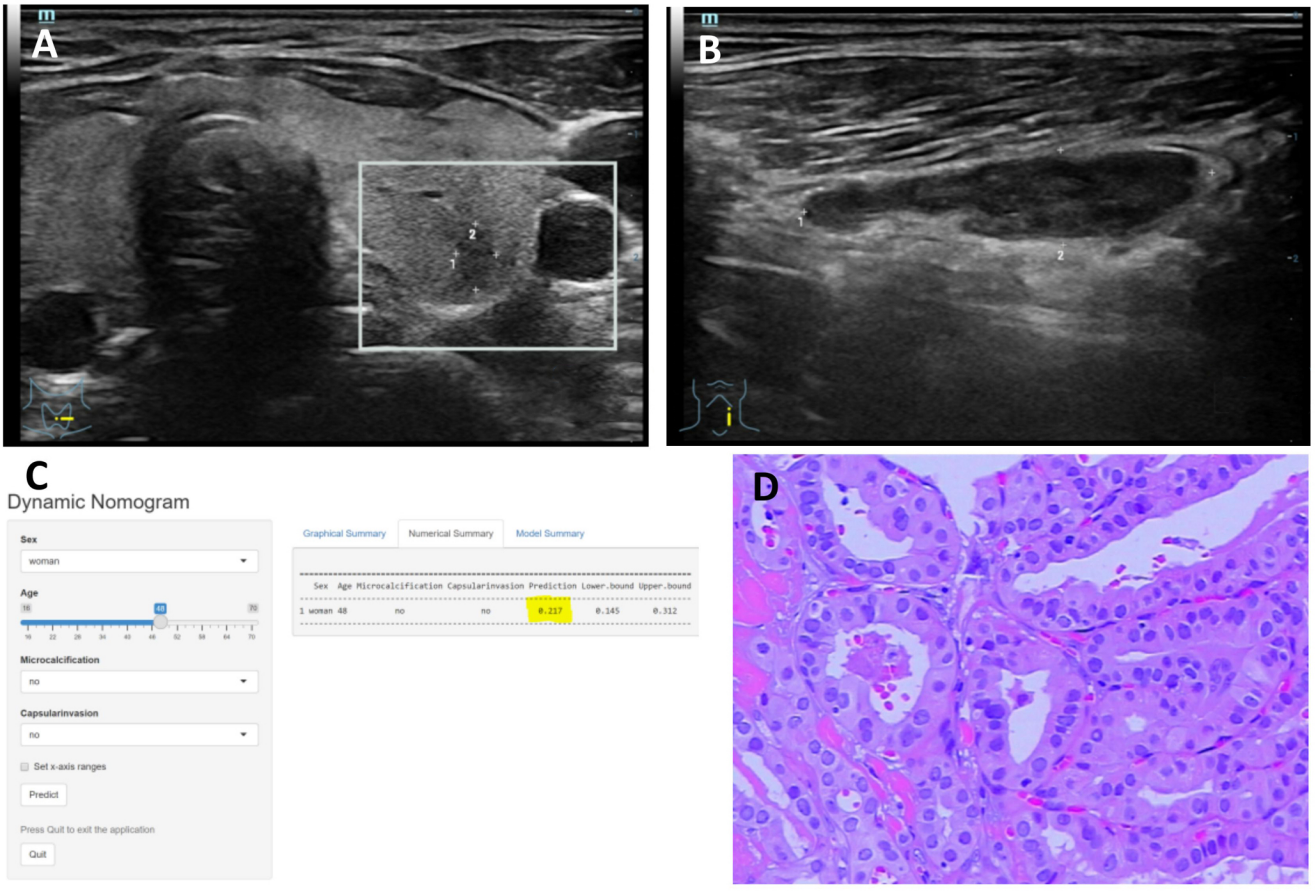
**(A–D)** Title: Representative example of dynamic web-based nomogram prediction for a 48-year-old female PTC patient without LNM. **(A)** US images showed the maximum diameter of hypoechoic thyroid nodule was 5.4mm in left lobe with no thyroid capsule invasion. **(B)** US images showed there was no microcalcification in the left lymph node. **(C)** Dynamic web-based nomogram predicted that the risk of LNM in this patient was 0.217. **(D)** Pathology showed PTC in left lobe with no thyroid capsule invasion and no LNM.

The DCA curve showed that the nomogram curve had a higher net benefit than the extreme curve and other ultrasound indexes, suggesting that the model had a good clinical application value for the occurrence of LNM in PTC. The clinical impact curve of this study suggested that the nomogram model had good clinical practicability (**Figure 4**). Representative examples of dynamic web-based nomogram prediction for LNM in PTC patients are shown in **Figures 5**, **6**.

## 4 Discussion

PTC is among the most frequently occurring types of cancer (11). While the mortality rate of PTC is not high, LNM significantly impacts the patient prognosis. Early detection of lymph node metastasis allows for lymph node dissection, which improves patient survival rates (12). If there is no LNM, unnecessary operations can be reduced. In more extensive surgery, interventions are influenced by more severe complications. Complications may arise in a significant number of patients, even in the most skilled hands and in high-volume centers (13). Postoperative hypocalcemia is observed in up to one third of thyroidectomy patients (14). There are other complications, such as hypoparathyroidism, injury to the recurrent laryngeal nerve, cervical hematoma, and wound infection (13, 15). These complications result in a decrease in the patients’ quality of life and increased costs for healthcare systems (13).

The present study identified nine variables, including gender, age, size, hypoechoic halo around the nodule, invasion of the thyroid capsule, microcalcification in lymph nodes, hyperechoic change in lymph nodes, PI, and AUC, which are associated with a high volume of CLNM in PTMC patients during the binary univariate logistic regression analysis.

While the incidence of papillary thyroid cancer is higher in women compared to men, men have a great risk of LNM than women in this type of cancer (16). Currently, the specific mechanism is unknown, but relevant studies suggested that it may be related to gender genes and hormone (17). Although some studies have shown that BRAF mutation and estrogen were not equally distributed between the sexes, whether they affect the occurrence of LNM in PTC needs to be further confirmed (18). Men have a higher basal metabolic rate compared to women, leading to accelerated tumor cell growth. Consequently, men face a higher risk for thyroid capsule invasion and LNM.

Previous studies used the TNM stage of AJCC thyroid cancer was used as a reference, with a cut-off age of 45 or 55 years (19). However, some scholars argue that age is negatively correlated with LNM in PTC and that there is no optimal cut-off value for age (20). While the incidence of PTC increases with age, the rate of LNM decreases (21). Our hypothesis is that the younger individuals have higher the metabolic rate and provide a more favorable environment for tumor cells. Therefore, younger patients with PTC are more likely to have LNM than older patients with PTC. In this study, age was included in the quantitative measures. The prediction model could give higher accurate individual LNM risk probability for each patient at different ages. Chan et al. (22). found that a distance from the capsule < 1.9 mm was associated with CLNM in cN0 PTC patients. A Meta-Analysis of clinicopathological significance of PTC located in the isthmus showed that isthmus PTC was associated with an increased risk of central LN metastasis (23). We conclude this for two reasons: first, the closer the lesion is to the thyroid capsule, the shorter the path for cancer cells to metastasize through the lymph nodes; second, the faster the cancer cells grow, the easier it is for them to invade the thyroid capsule, indicating that the malignant biological behavior of cancer cells is active.

Microcalcification is usually defined as calcification less than 1mm in diameter, which is a characteristic feature of PTC (24). This may be attributed to tumor thrombus from lymphatic or blood vessels. Or the glycoproteins and mucopolysaccharides produced by cancer cells leading to the formation of microcalcifications (25). The metastatic lymph nodes may have the characteristic features of the primary lesion, so it is inferred that the microcalcification in lymph nodes may originate from PTC. It has been demonstrated that hyperechoic changes in lymph nodes are caused by aggregates of thyroglobulin. Metastatic cancer cells are able to synthesize thyroglobulin but they lack an intact follicular structure. The synthesized thyroglobulin cannot be utilized and clumps form, presenting as hyperechoic change (26).

Zhang et al. (27) found that the maximum diameter of the nodule was the most important variable based on the study of 337 patients who had undergone the surgery on lymph node metastasis of PTC. Xue et al. (28) showed in their study that the risk of CLNM is greater when tumor size ≥1 cm. The maximum diameter of the nodule is related to tumor growth and proliferation, indicating tumors are more aggressive and prone to lymph node metastasis. The larger the nodule, the closer it is to the thyroid capsule. Larger nodules are prone to lymph node metastasis.

The hypoechoic halo may result from a progressing desmoplastic reaction or fibrosis of the capsule after the presence of microscopic capsular invasion (29). Some previous studies showed that the halo around thyroid nodules was caused by the rapid growth of the lesions and the extrusion of surrounding tissues (30). Our study showed that there was significant differences between LNM and non-LNM groups in terms of hypoechoic halo around the nodule. Hypoechoic halo may be associated with LNM, but it’s not a risk factor highly associated with LNM in PTC. This result corresponds with the relevant literature (31–33). We speculate that the appearance of hypoechoic halo indicates the malignant biological behavior of PTC cancer cells, which means that the tumor has a relatively strong ability to grow and infiltrate, which may be an important reason for its association with lymph node metastasis.

PI means maximum dose of microbubbles filling the ROI within a certain period of time, and AUC means total dose of microbubbles filling the ROI within a certain period of time, compared with those of adjacent tissue (34). Fast growing tumors are easy to ischemic necrosis and lack of nourishing blood vessels. The blood vessels of the high malignant tumors are messy and tortuous, which are easily blocked by tiny emboli and lead to stenosis (35). Therefore, the low values of PI and AUC have reference significance in predicting LNM of PTC.

The prediction model was developed through univariate and multivariate logistic regression analyses. And the model was evaluated and validated by ROC, calibration curve, cross-validation, DCA, and clinical impact curve. The results showed the prediction model was good and had favorable clinical practicability. We applied and registered our nomogram prediction model online. This model can be used to predict LNM through mobile phone, computer and other network login, which is convenient for clinical application.

Despite the predictive ability of our nomogram, there are still several limitations. Firstly, the number of cases for qualitative and quantitative CEUS analysis was insufficient. Therefore, the sample size can be expanded in the future. Secondly, indicators of gene mutations associated with PTC, such as BRAFV600E mutation (36) should be also involved in future analyses. The model can be further improved by incorporating ultrasound elastography, CT and other imaging techniques, as well as computer-aided diagnosis, to enhance its diagnostic ability.

## 5 Conclusion

Our study identified that male sex, younger age, hypoechoic halo around the nodule, thyroid capsule invasion, lymph node microcalcification, lymph node hyperechoic change, PI and AUC are associated with an increased risk of LNM in patients with PTC. Nomogram based on sex, age, thyroid capsule invasion, lymph node microcalcification has the potential for preoperative LNM risk assessment. The nomogram serves as a valuable clinical tool for aiding in clinical decision-making. It conveniently provides for individual preoperative prediction and might potentially improve the survival outcome in PTC patients with LNM.

## Data availability statement

The original contributions presented in the study are included in the article/supplementary material. Further inquiries can be directed to the corresponding author.

## Ethics statement

The studies involving humans were approved by Ethics Committee of Xiangya Hospital (No. 202206140). The studies were conducted in accordance with the local legislation and institutional requirements. The participants provided their written informed consent to participate in this study.

## Author contributions

JuL: Conceptualization, Data curation, Formal Analysis, Investigation, Methodology, Resources, Software, Visualization, Writing – original draft, Writing – review & editing. JinL: Conceptualization, Data curation, Investigation, Project administration, Resources, Supervision, Validation, Writing – review & editing. YC: Data curation, Formal Analysis, Resources, Writing – original draft. JiL: Data curation, Resources, Software, Writing – original draft. XH: Data curation, Resources, Writing – original draft. HZ: Data curation, Formal Analysis, Supervision, Writing – review & editing. BZ: Conceptualization, Data curation, Formal Analysis, Funding acquisition, Investigation, Methodology, Project administration, Resources, Supervision, Validation, Writing – review & editing, Writing – original draft.
